# Enhanced pentylenetetrazole sensitivity in a *C. elegans* mutant associated with *DNM1* encephalopathy

**DOI:** 10.17912/micropub.biology.000296

**Published:** 2020-08-24

**Authors:** Madeline A. Vaji, Guy A. Caldwell, Kim A. Caldwell

**Affiliations:** 1 Department of Biological Sciences, The University of Alabama, Tuscaloosa, AL 35487-0344

**Figure 1. Modeling convulsions associated with human DNM1 encephalopathy:**
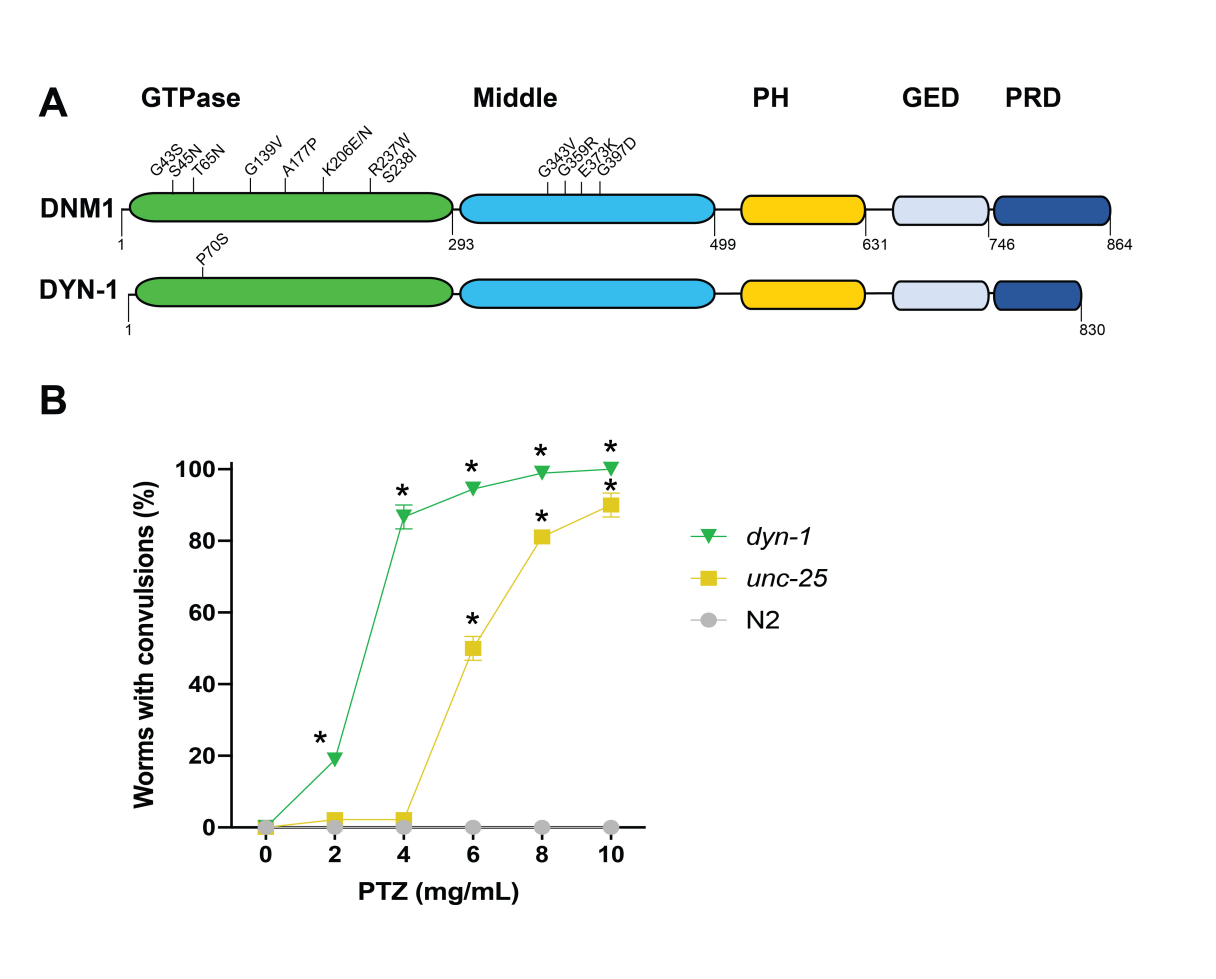
(A) Schematic comparison of human DNM1 and *C. elegans* DYN-1. The domains present in the proteins are shown and include the GTPase domain which is involved in GTP binding and hydrolysis, the middle domain, a GTPase effector domain (GED) required for oligomerization and stimulation of GTPase activity, the pleckstrin homology (PH) domain, and a proline-rich domain (PRD). Mutations associated with seizures in humans are indicated in the GTPase and middle domains of *DNM1*. A temperature-sensitive missense mutation in *C. elegans*, *dyn-1(ky51)*, is located in the GTPase domain. (B) PTZ induces convulsions in *C. elegans dyn-1(ky51)* and a positive control mutant, *unc-25(e156)*, in a dose-responsive manner, while N2 worms do not respond to this chemical. *p<0.0001 Two-Way ANOVA with a Dunnett’s post-hoc analysis where *dyn-1(ky51)* is compared to N2 and *unc-25(e156)* is compared to N2.

## Description

*DNM1* encephalopathy in humans is a neurological disorder that causes severe epilepsy with mutations in the gene *DNM1* (dynamin 1). This syndrome begins in childhood and persists through adulthood, requiring life-long management of severe seizures. The underlying cause of the seizures is not well understood. However, missense mutations associated with *DNM1* encephalopathy are clustered within either the GTPase domain or the middle domain, which, along with the GTPase effector domain (GED), is required for oligomerization and stimulation of GTPase activity (Fig. 1A) (Von Spiczak *et al.*, 2017). The nematode, *Caenorhabditis elegans,* is a proven model organism for studying epilepsy due to the ability to induce epileptic-like convulsions when mutant animals are treated with pentylenetetrazole (PTZ) (Williams *et al.*, 2004). *C. elegans* possess a homolog of human *DNM1*, termed *dyn-1*. Notably, there is a mutant, *dyn-1(ky51),* with a missense substitution (P70S) in the GTPase domain (Fig. 1A) (Clark *et al.*, 1997). The nematode proline residue is conserved in human DNM1 (P68). Through detailed analyses in multiple organisms, dynamin 1 has been characterized to function in endocytosis and synaptic transmission (Ferguson and De Camilli, 2012). In this context, we hypothesized that we could induce epileptic-like convulsions in *C. elegans* *dyn-1(ky51)* animals following treatment with the drug PTZ. Accordingly, we observed a dose-dependent convulsive effect in *dyn-1(ky51)* animals (Fig. 1B). These convulsions were similar to the positive control *unc-25(e156)*. UNC-25 is an ortholog of human glutamate decarboxylase and is involved in GABAergic synaptic transmission. The *dyn-1* animals exhibit a singular phenotype following exposure to PTZ; they displayed posterior paralysis with repetitive anterior convulsions at all tested concentrations, a phenotype we termed “head bobbing” that we associate with a tonic-clonic convulsion phenotype (Williams *et al.*, 2004). Furthermore, the *dyn-1* animals displayed the tonic-clonic phenotype almost immediately upon exposure to PTZ. The *unc-25* worms, however, displayed two different convulsion phenotypes following PTZ exposure. When exposed to 8 and 10 mg/mL PTZ, these animals also displayed head bobbing (tonic-clonic) convulsions and they occurred fairly quickly upon exposure to the drug. However, at a lower concentration, 6 mg/mL of PTZ, the phenotype of *unc-25* worms was a mixture of both heading bobbing (tonic-clonic) and full body paralysis (tonic convulsions) and it took longer for these phenotypes to appear (40 minutes on average); in this scenario the animal is stiff and will not respond to stimulus, but is still viable, as evidenced by pharyngeal activity. In summary, while *dyn-1(ky51)* animals displayed a consistent and immediate convulsion phenotype regardless of the concentration, phenotypes for *unc-25(e156)* worms were dependent on PTZ exposure concentration. It cannot be ruled out that the convulsive differences observed between these strains arise because different temperatures were used to raise the *dyn-1* animals (16°C) vs. the *unc-25* (20^o^C) animals.

## Methods

The *dyn-1(ky51)* mutation is a temperature-sensitive missense mutation that expresses wild-type dynamin at temperatures lower than 16°C, but within 30 minutes of exposure to 25°C, misfolded and non-functional protein is produced (Clark *et al.*, 1997). When animals reached the L4 stage, they were shifted from the permissive temperature (16°C) to the restrictive temperature (25°C) and examined for convulsions on PTZ-containing Petri dishes. N2 and *unc-25* worms were grown at 20°C until they also reached the L4 stage and were exposed to PTZ-containing plates.

NGM agar plates were made 48 hours before use and stored in a 20^o^C incubator. On the day of analysis, concentrations of 2-10 mg/mL PTZ (Sigma) were spread on top of the NGM plates using a cell spreader/hockey stick and dried for 60 minutes in a sterile hood with the lids ajar; solvent plates containing 0 mg/mL PTZ as “water only” controls were also prepared. Since the experiments were performed in triplicate, three plates per concentration/strain were utilized. The PTZ plates were then seeded with *E. coli* OP50, placed in the center of each plate, which was then allowed to dry for 30 minutes, with the lids ajar, in a sterile hood.

For each strain, 30 animals were then transferred to each plate and examined for convulsion activity for up to 60 minutes. When a worm displayed a convulsion, the phenotype was noted and the worm removed from the plate. Each strain was examined in triplicate, with 30 animals examined on each plate, for a total of 90 animals. Statistical analysis was performed with a Two-Way ANOVA with a Dunnett’s *post-hoc* analysis (GraphPad).

## Reagents

*C. elegans* strain CB156 *unc-25(e156)*

*C. elegans* strain CX51 *dyn-1(ky51)*

*E. coli* strain OP50 (saturated culture, previously grown in LB and stored at 4°C)

Pentylenetetrazole (Sigma)

Cell spreader/hockey stick (Thomas Scientific, 7012Q52)
